# Body Circumference and Cognitive Function: Role of Apolipoprotein E ε4 in the Elderly

**DOI:** 10.3390/ijms26125831

**Published:** 2025-06-18

**Authors:** Ji-Hyun Kim, Young Min Choe, Hye Ji Choi, Boung Chul Lee, Guk-Hee Suh, Shin Gyeom Kim, Hyun Soo Kim, Jaeuk Hwang, Dahyun Yi, Jee Wook Kim

**Affiliations:** 1 Department of Neuropsychiatry, Hallym University Dongtan Sacred Heart Hospital, Hwaseong 18450, Gyeonggi, Republic of Korea; jihyun.kim@hallym.or.kr (J.-H.K.); ymchoe@hallym.or.kr (Y.M.C.); phahyethon@hanmail.net (H.J.C.); suhgh@chol.com (G.-H.S.); 2 Department of Psychiatry, Hallym University College of Medicine, Chuncheon 24252, Gangwon, Republic of Korea; leeboungchul@hallym.or.kr; 3 Department of Neuropsychiatry, Hallym University Hangang Sacred Heart Hospital, Seoul 07247, Republic of Korea; 4 Department of Neuropsychiatry, Soonchunhyang University Bucheon Hospital, Bucheon 14584, Gyeonggi, Republic of Korea; redmensch@schmc.ac.kr; 5 Department of Laboratory Medicine, Hallym University Dongtan Sacred Heart Hospital, Hwaseong 18450, Gyeonggi, Republic of Korea; kimhyun@hallym.or.kr; 6 Department of Psychiatry, Soonchunhyang University Hospital, Seoul 04401, Republic of Korea; hju75@schmc.ac.kr; 7 Institute of Human Behavioral Medicine, Medical Research Center Seoul National University, Seoul 03080, Republic of Korea; dahyunyi@gmail.com

**Keywords:** mid-arm circumference, calf circumference, cognition, apolipoprotein E ε4 allele, Alzheimer’s disease

## Abstract

This study examined the relationships between mid-arm circumference (MAC) and calf circumference (CC) with cognitive performance, considering the moderating effect of apolipoprotein E ε4 allele (*APOE4*) status. Data from 196 older adults (65–90 years) in the General Lifestyle and AD (GLAD) study were analyzed. Cognitive performance was assessed using the CERAD neuropsychological battery, with episodic memory score (EMS) and non-memory score (NMS) as primary outcomes. Multiple linear regression analyses examined associations between MAC, CC, and waist circumference (WC) with cognition, adjusting for key covariates. Interaction effects with *APOE4* status were also explored. Higher MAC (or MAC/WC) significantly correlated with better EMS, while higher CC (or CC/WC) correlated with better NMS, even after Bonferroni correction (*P_B_* < 0.0125). These associations were stronger in *APOE4*-negative individuals but not significant in *APOE4*-positive participants. WC was not associated with cognitive measures. The results suggest that Upper- and lower-limb musculature may play distinct roles in cognitive function, with MAC linked to episodic memory and CC to non-memory cognition, particularly in *APOE4*-negative individuals. These findings highlight the potential of muscle health maintenance as a strategy for preserving cognitive function in aging populations.

## 1. Introduction

Age-related cognitive decline and neurodegenerative disorders, such as Alzheimer’s disease (AD), have been linked to various modifiable lifestyle and metabolic factors, including muscle mass, physical activity, and metabolic health [1,2,3,4]. While prior research has extensively examined the impact of obesity and overall body composition on cognitive function [5,6,7], less attention has been given to how specific muscle-related anthropometric measures, such as mid-arm and calf circumferences, contribute to different cognitive domains. The differential roles of upper- and lower-limb musculature in cognition remain poorly understood despite evidence suggesting that muscle mass distribution may influence distinct aspects of cognitive function.

Sarcopenia, the age-related loss of muscle mass and function, has been associated with increased cognitive decline and dementia risk [8,9]. However, different muscle groups may exert distinct effects on cognitive domains. The upper-limb musculature, particularly mid-arm muscles, may serve as a more accurate indicator of overall muscle reserve and systemic metabolic health, potentially influencing episodic memory [10]. In contrast, the lower limb musculature, particularly the calf muscles, is primarily involved in mobility, circulation, and postural stability, which are essential for executive function and attention [11]. Given these roles, mid-arm circumference (MAC) and calf circumference (CC) may serve as distinct yet complementary markers for cognitive health.

Furthermore, body composition does not act in isolation but interacts with genetic risk factors, particularly the apolipoprotein E ε4 allele (*APOE4*), which is the strongest genetic risk factor for late-onset AD [12]. *APOE4* carriers exhibit higher central adiposity, increased neuroinflammatory responses, and altered lipid metabolism, which may impair the neuroprotective effects of muscle mass [13,14,15]. Prior studies have suggested that APOE4 carriers experience greater metabolic dysregulation and oxidative stress [13,14,15], yet few have investigated whether the relationship between muscle mass and cognitive performance differs by *APOE4* status.

This study was conducted to examine the relationship of MAC and CC with cognitive performance in non-demented older adults while evaluating how these associations differ across cognitive domains and *APOE4* status. Based on the distinct roles of upper- and lower-limb musculature, we hypothesize that MAC will be more strongly associated with episodic memory, whereas CC will be more related to non-memory cognitive functions, such as executive function and attention. Additionally, we explore whether these associations are modified by *APOE4* status, given the genotype’s known impact on systemic metabolism and neuroinflammation [13,14,15]. Waist circumference (WC), which primarily reflects central adiposity rather than muscle mass, was included as a secondary factor to further contextualize body composition effects.

## 2. Results

### 2.1. Participant Characteristics

The demographic and clinical characteristics of the study population, comprising 156 *APOE4*-negative and 40 *APOE4*-positive participants, are summarized in Table 1. Differences in body circumference measurements between these groups were observed, while no significant differences were noted for other demographic or clinical variables.

### 2.2. Association of Body Circumference with Cognition

**Figure 1:**
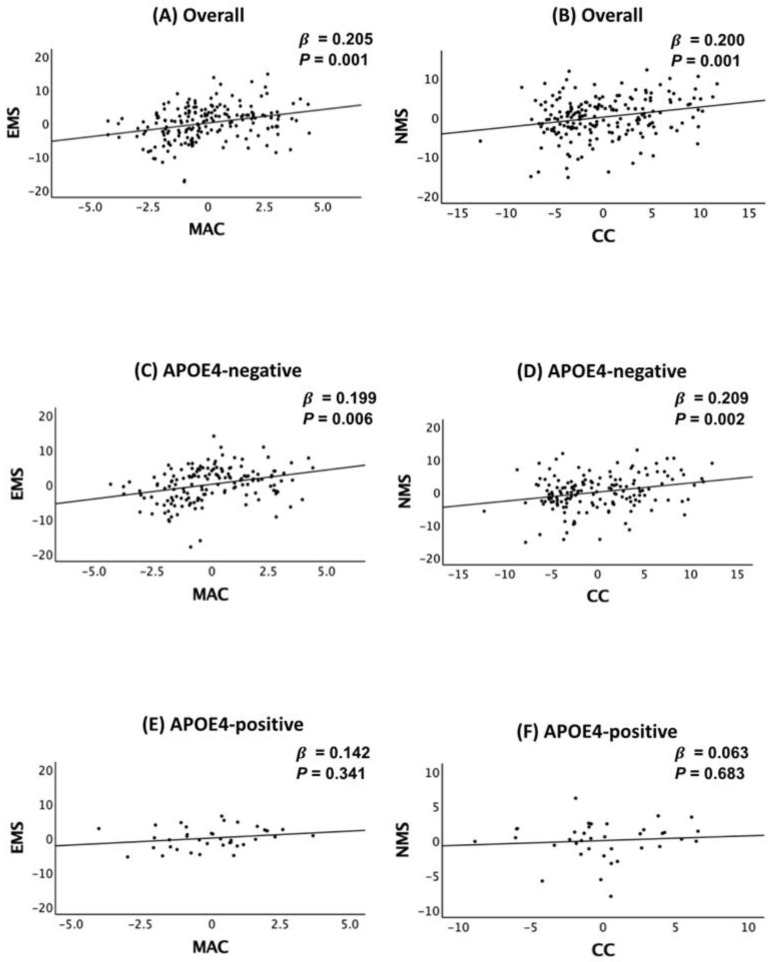
Partial regression plot for the association between mid-arm circumference (MAC) and episodic memory score (EMS) (**A**,**C**,**E**) and between calf circumference (CC) and non-memory score (NMS) (**B**,**D**,**F**) in non-demented older adults according to the *APOE4* status. Abbreviations: CERAD, the Consortium to Establish a Registry for Alzheimer’s Disease; *APOE4*, apolipoprotein ε4 allele. Multiple linear regression analyses were performed after adjusting for all confounders.

A greater MAC (or MAC/WC) was significantly associated with higher EMS, while a greater CC (or CC/WC) was significantly associated with higher NMS (Table 2 and Figure 1A,B). These associations remained significant even after applying the Bonferroni correction (*P_B_* < 0.0125), suggesting distinct roles for upper- and lower-limb musculature in cognitive performance. In contrast, WC and MAC/CC showed no significant association with any cognitive measures (Table 2).

### 2.3. APOE4 Moderation of the Association Between Body Circumference and Cognition

*APOE4* status moderated the associations between MAC (or MAC/WC) and EMS, as well as between CC (or CC/WC) and NMS (Table 3). MAC (or MAC/WC) was significantly associated with EMS in *APOE4*-negative participants, but this effect was not observed in *APOE4*-positive individuals. Similarly, the association between CC and NMS was significant in *APOE4*-negative participants, whereas no such relationship was found in *APOE4*-positive individuals (Table 4 and Figure 1C–F).

### 2.4. Sensitivity Analyses

Sensitivity analyses conducted on participants without recent reductions in food intake supported the robustness of the primary findings. The associations between MAC (or MAC/WC) with EMS and CC (or CC/WC) with NMS remained significant, reinforcing distinct roles for body musculature in cognitive performance (Table 5).

## 3. Discussion

This study highlights a significant association between body circumferences and cognitive function, demonstrating that MAC is primarily linked to EMS, while CC is more associated with NMS. These findings suggest that upper- and lower-limb musculature play distinct roles in cognitive performance. Furthermore, these associations were more pronounced in *APOE4*-negative individuals, underscoring the potential moderating effect of *APOE4* status on the relationship between muscle mass distribution and cognitive function.

A key contribution of this study is its differentiation between upper- and lower-limb musculature in relation to specific cognitive domains. While previous research has broadly linked overall musculature to cognition [1,5,6,7,16,17,18], our findings refine this relationship by showing that upper-limb musculature (i.e., MAC) is more strongly associated with memory-related processes, whereas lower-limb musculature (i.e., CC) is more relevant for non-memory cognitive functions, such as executive function and attention. These results highlight the importance of muscle distribution rather than total musculature in maintaining cognitive resilience.

This differentiation supports the hypothesis that MAC, as a proxy for upper-limb musculature, is associated with memory-related cognitive processes. Specifically, MAC may serve as a surrogate marker for overall muscle reserve, which is associated with reduced inflammation, improved metabolic health, and enhanced neurotrophic support, including increased secretion of neurotrophic factors such as Brain-Derived Neurotrophic Factor (BDNF), which are known to support hippocampal integrity and episodic memory enhancement [19,20]. Moreover, in older adults, greater MAC may reflect a preserved physical capacity to engage in cognitively enriching manual activities—such as gardening, knitting, or playing musical instruments—which involve upper-limb use and environmental interaction. Although MAC itself does not measure fine motor coordination, reduced upper-limb muscle mass may limit access to these activities, thereby decreasing opportunities for cognitive stimulation. Accordingly, the association between MAC and episodic memory may be partially mediated by increased participation in such activities, along with underlying systemic health advantages [21,22,23].

Conversely, CC, as a proxy for lower-limb musculature, is crucial for supporting non-memory cognitive functions such as executive functions and attention, facilitating mobility, balance, and circulation. These activities bolster frontostriatal and cerebellar pathways, which are essential for cognitive processes [11,24]. Aerobic exercises involving the lower limbs not only enhance cardiovascular health but also increase cerebral blood flow, primarily through the ‘peripheral pump’ action of the calf muscles, which improves venous return and cerebral perfusion, benefiting prefrontal cortex function [25,26]. Furthermore, the strength and mass of lower-limb muscles (measured by leg power) are associated with cognitive changes over ten years and contribute to the preservation of total gray matter [27]. Moreover, aerobic exercise and resistance training, including the lower body, can significantly enhance executive functions such as selective attention, alongside notable improvements in gait speed [28,29,30]. However, reduced CC in the lower limbs could lead to cerebral hypoperfusion, adversely affecting executive function and attentional control [31]. Consequently, while lower-limb exercises generally boost overall brain function, their primary impact is on enhancing neural efficiency and plasticity in networks within the prefrontal cortex that govern executive functions and attention [32,33] rather than directly influencing memory-centric regions like the hippocampus.

Our findings indicate that the *APOE4* status moderates the link between upper- and lower-limb musculature and cognitive function. This reduction in neuroprotection could explain the null associations between musculature and cognition observed in *APOE4* carriers. *APOE4* is linked to higher levels of neuroinflammatory cytokines and oxidative stress [34,35], further impairing the benefits of myokines like BDNF. Supporting this, a preclinical study showed that *APOE4* diminishes BDNF expression by promoting the nuclear translocation of histone deacetylases (HDACs) in human neurons [36]. Furthermore, *APOE4* carriers are at greater risk for cerebral small vessel disease and reduced cerebral blood flow [37,38], which disproportionately affects executive function and attention. These findings suggest that the metabolic and inflammatory profiles associated with *APOE4* adversely affect the cognitive benefits that typically arise from healthy muscle mass.

Despite its strengths, this study has several limitations. First, the cross-sectional design limits causal inference, restricting conclusions about the temporal and mechanistic links between muscle mass and cognitive function. Nonetheless, such designs are valuable for identifying novel, clinically relevant associations and generating hypotheses, particularly in aging populations where early risk detection is critical. The domain-specific associations observed in this study offer a conceptual basis for future longitudinal and interventional research to clarify these relationships. Second, although MAC and CC serve as established proxies for muscle mass [39], especially in geriatric populations [40], they do not directly quantify muscle composition or functional capacity [41]. In this regard, the use of technology-based measurements—such as dual-energy X-ray absorptiometry (DEXA) [42], bioelectrical impedance analysis (BIA) [43], or magnetic resonance imaging (MRI) [44]—might be recommended for improved accuracy in future studies, when they are available. In addition, strength-based assessments—particularly grip strength—should be incorporated, as they provide a clinically validated measure of muscle function and are considered a reliable index of cognitive decline [45]. Combining both structural and functional indicators would allow for a more comprehensive evaluation of muscle health in relation to cognitive outcomes, especially considering their strong correlation and the potential discordance—given that declines in muscular strength often precede reductions in muscle mass [39,46]. Third, the limited number of *APOE4*-positive participants (*n* = 40) constrained statistical power for subgroup analyses. Expanding the sample size is necessary to enhance the reliability of findings on genetic influences in the relationship between muscle mass and cognition. Fourth, adiposity may influence anthropometric measurements, potentially affecting their accuracy in estimating muscle mass. However, this study accounted for such concerns by analyzing MAC and CC relative to WC (MAC/WC and CC/WC), which produced consistent results, reducing the likelihood of adiposity-driven bias. Furthermore, given the participants’ mean BMI of 24.83 kg/m^2^ (SD = 3.41) and a low prevalence of obesity (6.12%, *n* = 12, defined as BMI ≥ 30 kg/m^2^), the impact of excess adiposity was likely minimal [47]. Additionally, previous research suggests that BMI adjustment enhances the predictive validity of anthropometric measures in assessing muscle mass and related outcomes [48]. To address potential confounding, this study included BMI as a covariate, and future work should further refine BMI-adjusted MAC and CC thresholds to improve their application in detecting sarcopenic obesity [47,48]. Fifth, although men and women differ in absolute muscle mass and cognitive performance, and previous studies have suggested potential sex-specific associations between upper- and lower-limb musculature and cognitive function [49,50], our findings indicate that these associations were consistent across sexes. To examine potential sex effects, we conducted interaction analyses and sex-stratified multiple regression models (Tables S1 and S2). No significant interaction between sex and regional muscle mass was observed, and the direction and magnitude of associations remained stable across both men and women. These results suggest that the relationship between regional muscle mass and cognitive function may operate similarly across sexes, despite underlying biological differences. This may reflect shared mechanisms—such as inflammation, vascular function, or physical activity—that contribute to muscle–brain interaction regardless of sex. These findings highlight the need for future research to investigate sex-specific patterns using diverse populations and direct measures of muscle mass.

Despite these limitations, anthropometric measures such as MAC and CC remain practical, non-invasive tools for monitoring muscle status over time due to their universal availability and ease of use [39]. Regular assessments using these measures may help identify individuals at risk of cognitive decline, supporting early interventions to preserve cognitive function in aging populations.

## 4. Materials and Methods

### 4.1. Participants

This study is part of the General Lifestyle and AD (GLAD) study, a prospective cohort study initiated in 2020. As of July 2022, the study enrolled 196 adults aged 65 to 90 years who were free of clinical dementia, including 83 cognitively normal (CN) individuals and 113 individuals with mild cognitive impairment (MCI). Recruitment was conducted through a dementia screening program at the memory clinic of Hallym University Dongtan Sacred Heart Hospital, Hwaseong, Gyeonggi, Republic of Korea. To complement clinic-based recruitment, additional participants were identified through community outreach efforts, including recommendations from previous participants, their families, or acquaintances. Careful selection ensured that the cohort represented the general population, with a focus on capturing a broad spectrum of cognitive health in older adults. Participants in the CN group were those with a Clinical Dementia Rating (CDR) [51] score of 0 and no history of MCI or dementia. Individuals with MCI were identified based on established amnestic MCI criteria, requiring informant-confirmed memory concerns, measurable memory deficits, preserved overall cognitive function, independence in daily living activities, and no evidence of dementia. Memory impairment was assessed using age-, education-, and sex-adjusted z-scores, with scores below –1.0 on at least one of four episodic memory assessments from the Korean version of the Consortium to Establish a Registry for Alzheimer’s Disease (CERAD) neuropsychological battery [52,53]: word list memory, word list recall, word list recognition, and the constructional recall [52,53,54]. All MCI participants had a CDR score of 0.5. Participants were excluded if they had significant psychiatric or neurological disorders, comorbidities affecting cognitive function, illiteracy, severe sensory deficits, difficulties with communication or behavior affecting clinical evaluation, or if they were taking experimental medications.

### 4.2. Standard Protocol Approvals, Registrations, and Participants Consent

The study protocol received approval from the Institutional Review Board of Hallym University Dongtan Sacred Heart Hospital and was carried out in compliance with the latest guidelines of the Declaration of Helsinki. Informed consent was obtained from all participants or their legal representatives.

### 4.3. Clinical Assessments

Participants underwent comprehensive clinical evaluations conducted by trained psychiatrists in accordance with the GLAD study’s standardized assessment protocol, which includes the CERAD clinical and neuropsychological battery [52,53]. Licensed psychologists with expertise in geriatric populations administered the CERAD neuropsychological battery [54]. All clinical evaluations and diagnoses were based on a consensus approach involving psychiatrists and psychologists with expertise in dementia. The assessments included measures of episodic memory, non-memory cognitive domains, and overall cognitive performance. Episodic memory decline, a hallmark early symptom of Alzheimer’s disease [55,56,57,58,59,60], was quantified using the episodic memory score (EMS), calculated by summing scores from four tasks in the CERAD neuropsychological battery: word list memory, word list recall, word list recognition, and constructional recall. The non-memory score (NMS) was calculated by adding the scores from three non-memory tests: verbal fluency (executive function/attention/language), the modified Boston Naming Test (language), and constructional praxis (visuospatial and perceptual abilities) [61]. Global cognition was assessed using the CERAD total score (TS) [62], derived by summing the results of seven tests: verbal fluency, modified Boston Naming Test, word list memory, constructional praxis, word list recall, word list recognition, and constructional recall. Vascular risks were evaluated during structured interviews conducted by trained researchers with participants and their family members. The vascular risk score (VRS), expressed as a percentage, was calculated by summing the presence of conditions such as hypertension, diabetes mellitus, dyslipidemia, coronary heart disease, transient ischemic attack, and stroke [63]. The body mass index (BMI) was calculated using weight in kilograms divided by height in meters squared, following the World Health Organization guidelines (https://iris.who.int/handle/10665/37003, accessed on 12 June 2025). The Geriatric Depression Scale (GDS) was employed to assess depressive symptoms among participants [64,65]. Economic status was classified into three tiers according to annual income relative to the minimum cost of living (MCL) set by the Ministry of Health and Welfare, Republic of Korea. The reference values, determined in November 2012 (http://www.law.go.kr, accessed on 12 June 2025), specified that for a single-person household, the MCL was 572,168 Korean Won (approximately US $507.9) per month, with an additional 286,840 Korean Won (approximately US $254.6) allocated per month for each extra household member. Physical activity levels were assessed using the Korean version of the Physical Activity Scale for the Elderly (PASE) [66,67], which evaluates the intensity, frequency, and duration of leisure-time activities, as well as household and occupational tasks performed over the past week. The total PASE score, derived from these components, provided a measure of overall physical activity. Higher scores indicated greater engagement in physical activities, whereas lower scores suggested reduced physical activity levels. Lifetime alcohol intake status (never/former/drinker) and lifetime smoking status (never/ex-smoker/smoker) were evaluated through trained researcher interviews and a medical record review. Nutritional assessments were performed using the Mini-Nutritional Assessment (MNA) tool [68], a validated instrument for evaluating nutrition in older adults. The MNA considered factors such as recent reductions in food intake due to appetite loss, digestive issues, or chewing/swallowing difficulties. To ensure the reliability of the data, informants were interviewed when necessary [68].

### 4.4. Measuring Body Circumferences and Blood Biomarkers

Anthropometric measurements were conducted following standardized protocols to assess regional muscle distribution and central adiposity, which may have distinct effects on cognitive function. MAC and CC were measured in accordance with the MNA guidelines, a validated instrument for evaluating nutritional status in older adults [69]. WC, which was not included in the MNA, was additionally assessed in this study to account for central adiposity and metabolic risk [70].

MAC was measured at the midpoint between the acromion process of the scapula and the olecranon process of the ulna, with the participant’s arm relaxed and hanging freely [69,71].

CC was measured at the widest part of the calf while the participant was in a seated position with the knee flexed at 90 degrees, reflecting lower-limb muscle mass and mobility-related function [69,72].

WC was measured at the midpoint between the lower rib margin and the iliac crest using a flexible, non-stretchable measuring tape, ensuring that the tape was parallel to the floor, as recommended for central adiposity assessment [70].

Each measurement was taken twice, and the average was recorded to minimize measurement error.

Blood Biomarker Analysis Blood samples were collected in the morning (8–9 A.M.) via venipuncture and placed in trace element-free tubes to avoid contamination. Serum albumin, glucose, high-density lipoprotein (HDL)-cholesterol, and low-density lipoprotein (LDL)-cholesterol were measured using the COBAS c702 analyzer with dedicated reagents supplied by Roche Diagnostics (Mannheim, Germany). All biochemical analyses were performed in a centralized laboratory under strict quality control procedures to ensure accuracy and reproducibility.

### 4.5. APOE4 Genotyping

Blood samples were obtained using vacutainer tubes containing EDTA as an anticoagulant (BD Vacutainer^®^, Becton, Dickinson and Company, Franklin Lakes, NJ, USA). DNA was isolated with the QIAamp DSP DNA Blood Mini Kit, supported by the QIAcube HT System (QIAGEN, Hilden, Germany; https://www.qiagen.com). Genotyping for APOE was conducted using the Seeplex ApoE ACE Genotyping Kit (Seegene, Seoul, Republic of Korea; https://www.seegene.com) on a ProFlex PCR system (ThermoFisher Scientific, Waltham, MA, USA; https://www.thermofisher.com), following the provided instructions. After amplification, PCR products underwent analysis with the QIAxcel Advanced System (QIAGEN, Hilden, Germany; https://www.qiagen.com), a capillary electrophoresis device, and genotypes were classified as ε2/ε2, ε2/ε3, ε2/ε4, ε3/ε3, ε3/ε4, or ε4/ε4 based on electrophoretic patterns and manufacturer guidelines. The presence of one or more ε4 alleles indicated *APOE4*-positivity.

### 4.6. Statistical Analysis

The association between body circumference measurements and cognitive performance was evaluated using multiple linear regression analyses. MAC, CC, and WC were treated as continuous independent variables, while EMS, NMS, and TS served as dependent variables.

To account for potential confounders, multiple covariates were included, such as age, sex, *APOE4* status, education, clinical diagnosis, vascular risk factors, BMI, depression, physical activity, annual income, alcohol intake, smoking, and blood markers (albumin, glucose, HDL- cholesterol, and LDL-cholesterol levels). Two stepwise models were applied: Model 1: Adjusted for age, sex, *APOE4* status, education, clinical diagnosis, vascular risk factors, and BMI. Model 2: Further incorporated GDS score, PASE score, annual income, lifetime alcohol intake status, lifetime smoking status, albumin, glucose, HDL-cholesterol, and LDL cholesterol levels to explore potential metabolic contributions. For each type of analysis, a Bonferroni-corrected *p* (*P_B_* = 0.05/number of analyses) was applied as the threshold for statistical significance; the *P_B_* was < 0.0125 (0.05/4).

To examine whether *APOE4* status moderated the relationship between body circumferences and cognitive performance, multiple linear regression models were used, incorporating two-way interaction terms between body circumference variables and cognition as independent variables. When significant interactions were detected, stratified analyses were conducted separately for *APOE4*-positive and *APOE4*-negative groups to explore genotype-specific effects.

Sensitivity analyses were performed on individuals without reduced food intake in the past three months to control for possible confounding effects of physical or mental health conditions on body circumference and cognitive performance. All statistical analyses were performed using SPSS Statistics software version 28 (IBM, Armonk, NY, USA).

All statistical analyses were performed using SPSS Statistics software version 28 (IBM, Armonk, NY, USA).

## 5. Conclusions

This study highlights distinct associations between muscle-related body circumferences and cognitive function, with MAC correlating more strongly with episodic memory and CC with non-memory cognitive domains. Notably, these relationships were more pronounced in *APOE4*-negative individuals, implying that muscle preservation may offer greater cognitive benefits in those without a genetic predisposition to AD. Moving forward, longitudinal studies and advanced imaging techniques are needed to further elucidate the mechanisms underlying these associations. Moreover, interventions targeting both upper- and lower-limb muscle maintenance could serve as a promising approach to mitigating cognitive decline, reinforcing the importance of muscle health in aging populations.

## Figures and Tables

**Table 1:** Clinical characteristics of the participants according to *APOE4* status.

	Overall	*APOE4*-Negative	*APOE4*-Positive	*p*
n	196	156	40	
Age, y	72.65 (5.95)	72.95 (5.96)	71.50 (5.86)	0.170 ^a^
Female, n (%)	138 (70.41)	106 (67.95)	32 (80.00)	0.136 ^b^
Education, y	9.62 (4.51)	9.61 (4.55)	9.68 (4.38)	0.934 ^a^
MCI, n (%)	113 (57.65)	88 (56.41)	25 (62.50)	0.487 ^b^
VRS, %	23.98 (18.58)	23.93 (19.14)	24.17 (16.43)	0.943 ^a^
MMSE	25.58 (3.45)	25.52 (3.46)	25.83 (3.43)	0.618 ^a^
Body circumference index				
Measures				
MAC, cm	25.66 (3.28)	25.60 (3.31)	25.90 (3.19)	0.612 ^a^
CC, cm	36.41 (5.18)	36.76 (5.18)	34.97 (5.00)	0.053 ^a^
WC, cm	87.98 (8.97)	87.98 (8.68)	87.95 (10.17)	0.983 ^a^
Ratio				
MAC/WC	0.29 (0.04)	0.29 (0.04)	0.30 (0.04)	0.535 ^a^
CC/WC	0.42 (0.05)	0.42 (0.05)	0.40 (0.06)	0.050 ^a^
MAC/CC	0.72 (0.13)	0.71 (0.13)	0.75 (0.13)	0.060 ^a^
Body mass index	24.83 (3.41)	24.78 (3.41)	25.03 (3.40)	0.680 ^a^
PASE	64.77 (46.21)	64.45 (47.19)	66.04 (42.70)	0.847 ^a^
Decrease in food intake over the past three months				1.00 ^c^
No, n (%)	182 (92.86)	145 (92.95)	37 (92.50)	
Yes, n (%)	14 (7.14)	37 (23.72)	3 (7.50)	
Serum nutritional markers				
Albumin, g/dL	4.57 (0.26)	4.57 (0.26)	4.60 (0.25)	0.465 ^a^
Glucose, fasting, mg/dL	108.15 (19.94)	108.46 (21.02)	106.87 (14.87)	0.660 ^a^
HDL-Cholesterol, mg/dL	54.64 (12.96)	54.51 (12.89)	55.21 (13.38)	0.765 ^a^
LDL-Cholesterol, mg/dL	96.41 (33.82)	96.10 (35.42)	97.68 (26.64)	0.796 ^a^
Cognition				
Memory score				
EMS	35.10 (9.48)	35.17 (9.47)	34.83 (9.67)	0.840 ^a^
Non-memory score				
NMS	34.25 (6.62)	34.06 (6.92)	35.00 (5.26)	0.423 ^a^
Global cognition				
TS	69.98 (15.61)	70.00 (16.15)	69.90 (13.52)	0.971 ^a^

Abbreviations: MAC, mid-arm circumference; CC, calf circumference; WC, waist circumference; *APOE4*, apolipoprotein E ε4 allele; MCI, mild cognitive impairment; VRS, vascular risk score; MMSE, mini-mental state examination; HDL, high-density lipoprotein; LDL, low-density lipoprotein; EMS, episodic memory score; NMS, non-memory score; TS, total score of the Consortium to Establish a Registry for Alzheimer’s Disease. Data are expressed as mean (standard deviation) unless otherwise indicated. ^a^ By student *t*-test. ^b^ By chi-square test. ^c^ By Fisher exact test.

**Table 2:** Results of the multiple linear regression analyses of the association between body circumference and cognition.

	MAC		CC		WC		MAC/WC		CC/WC		MAC/CC	
	β	*p*	β	*p*	β	*p*	β	*p*	β	*p*	β	*p*
EMS												
Model 1	0.196	0.004	−0.026	0.583	−0.131	0.023	0.167	<0.001	0.046	0.347	0.083	0.082
Model 2	0.205	0.001	−0.037	0.414	−0.084	0.139	0.148	0.002	0.005	0.911	0.095	0.040
NMS												
Model 1	0.106	0.249	0.194	0.002	−0.071	0.364	0.076	0.256	0.247	<0.001	0.083	0.082
Model 2	0.128	0.152	0.200	0.001	−0.031	0.692	0.069	0.296	0.225	<0.001	0.095	0.040
TS												
Model 1	0.155	0.041	0.032	0.540	−0.123	0.058	0.137	0.013	0.102	0.062	0.025	0.643
Model 2	0.177	0.016	0.026	0.614	−0.084	0.192	0.131	0.017	0.070	0.194	0.038	0.472

Abbreviations: MAC, mid-arm circumference; CC, calf circumference; WC, waist circumference; *APOE4*, apolipoprotein E ε4 allele; EMS, episodic memory score; TS, total score of the Consortium to Establish a Registry for Alzheimer’s Disease; VRS, vascular risk score. The first model included age, sex, *APOE4*, education, clinical diagnosis, VRS, and BMI as covariates; the second model included those covariates plus GDS score, PASE score, annual income, alcohol intake, smoking, albumin, fasting glucose, and HDL- or LDL-cholesterol.

**Table 3:** Results of multiple linear regression analyses that included interaction terms for the association between body circumference and *APOE4*-positivity in predicting cognition.

	MAC		CC		MAC/WC		CC/WC		MAC/CC	
	β	*p*	β	*p*	β	*p*	β	*p*	β	*p*
EMS										
BC	0.209	<0.001	−0.047	0.352	0.144	0.002	0.002	0.976	0.097	0.056
*APOE4*-positivity	0.206	0.005	−0.225	0.477	0.201	0.007	−0.132	0.680	−0.048	0.848
BC ×* APOE4*-positivity	−0.265	<0.001	0.145	0.647	−0.264	<0.001	0.055	0.863	−0.043	0.866
NMS										
BC	0.130	0.149	0.265	<0.001	0.065	0.330	0.282	<0.001	−0.117	0.093
*APOE4*-positivity	0.118	0.249	0.352	0.004	0.136	0.197	0.378	0.002	−0.093	0.788
BC ×* APOE4*-positivity	−0.122	0.245	−0.342	0.005	−0.142	0.185	−0.448	<0.001	0.102	0.769
TS										
BC	0.181	0.014	0.037	0.519	0.131	0.017	0.097	0.101	0.036	0.534
*APOE4*-positivity	0.122	0.149	0.113	0.754	0.122	0.157	0.347	0.336	−0.031	0.913
BC ×* APOE4*-positivity	−0.174	0.045	−0.164	0.648	−0.180	0.041	−0.396	0.271	−0.026	0.928

Abbreviations: BC, body circumference; MAC, mid-arm circumference; CC, calf circumference; WC, waist circumference; *APOE4*, apolipoprotein ε4 allele; EMS, episodic memory score; NMS, non-memory score; TS, total score of the Consortium to Establish a Registry for Alzheimer’s Disease. To explore the moderating effects of *APOE4*-positivity on the associations between BC and cognition, i.e., EMS, NMS, and TS, multiple linear regression analyses were performed, including two-way interaction terms between BC and cognition as additional independent variables.

**Table 4:** Results of the multiple linear regression analyses of the association between body circumference and cognition according to the *APOE4* subgroup.

	MAC		CC		MAC/WC		CC/WC		MAC/CC	
	β	*p*	β	*p*	β	*p*	β	*p*	β	*p*
EMS										
*APOE4*-negative										
Model 1	0.200	0.006	−0.041	0.421	0.185	<0.001	0.032	0.535	0.089	0.079
Model 2	0.199	0.006	−0.058	0.255	0.172	0.002	−0.008	0.883	0.106	0.036
*APOE4*-positive										
Model 1	0.170	0.418	0.093	0.501	0.058	0.667	0.134	0.384	−0.026	0.855
Model 2	0.142	0.341	−0.110	0.302	0.113	0.324	−0.108	0.388	0.100	0.326
NMS										
*APOE4*-negative										
Model 1	0.148	0.138	0.208	0.002	0.134	0.069	0.278	<0.001	−0.108	0.116
Model 2	0.172	0.086	0.209	0.002	0.140	0.064	0.260	<0.001	−0.103	0.139
*APOE4*-positive										
Model 1	−0.145	0.598	0.163	0.367	−0.215	0.223	0.083	0.683	−0.154	0.400
Model 2	−0.160	0.458	0.063	0.683	−0.276	0.087	−0.103	0.569	−0.094	0.528
TS										
*APOE4*-negative										
Model 1	0.173	0.033	0.032	0.571	0.177	0.003	0.114	0.046	0.027	0.630
Model 2	0.197	0.016	0.022	0.697	0.185	0.003	0.086	0.138	0.042	0.460
*APOE4*-positive										
Model 1	0.046	0.848	0.127	0.418	−0.081	0.597	0.084	0.631	−0.087	0.585
Model 2	−0.043	0.790	−0.041	0.722	−0.093	0.456	−0.130	0.334	−0.004	0.968

Abbreviations: MAC, mid-arm circumference; CC, calf circumference; WC, waist circumference; *APOE4*, apolipoprotein E ε4 allele; EMS, episodic memory score; TS, total score of the Consortium to Establish a Registry for Alzheimer’s Disease; VRS, vascular risk score. The first model included age, sex, *APOE4*, education, clinical diagnosis, VRS, and BMI as covariates; the second model included those covariates plus GDS score, PASE score, annual income, alcohol intake, smoking, albumin, fasting glucose, and HDL- or LDL-cholesterol.

**Table 5:** Results of the multiple linear regression analyses of the association between body circumference and cognition in old adults with no decrease in food intake over the past 3 months (*n* = 182).

	MAC		CC		MAC/WC		CC/WC		MAC/CC	
	β	*p*	β	*p*	β	*p*	β	*p*	β	*p*
EMS										
Overall										
Model 1	0.176	0.008	−0.041	0.383	0.158	0.001	0.029	0.554	0.083	0.080
Model 2	0.174	0.006	−0.046	0.320	0.127	0.009	−0.010	0.838	0.088	0.057
*APOE4*-negative										
Model 1	0.183	0.009	−0.060	0.228	0.177	0.001	0.009	0.866	0.098	0.049
Model 2	0.176	0.012	−0.073	0.150	0.158	0.005	−0.030	0.568	0.110	0.029
*APOE4*-positive										
Model 1	0.162	0.449	0.101	0.477	0.053	0.699	0.141	0.371	−0.035	0.810
Model 2	0.089	0.538	−0.100	0.327	0.092	0.390	−0.078	0.519	0.076	0.436
NMS										
Overall										
Model 1	0.064	0.487	0.199	0.002	0.066	0.343	0.265	<0.001	−0.130	0.048
Model 2	0.087	0.347	0.214	0.001	0.055	0.437	0.252	<0.001	−0.134	0.043
*APOE4*-negative										
Model 1	0.116	0.246	0.213	0.002	0.135	0.079	0.298	<0.001	−0.123	0.082
Model 2	0.142	0.167	0.225	0.002	0.145	0.078	0.294	<0.001	−0.123	0.092
*APOE4*-positive										
Model 1	−0.217	0.439	0.205	0.270	−0.252	0.152	0.116	0.578	−0.210	0.262
Model 2	−0.229	0.303	0.075	0.635	−0.298	0.063	−0.077	0.682	−0.122	0.420
TS										
Overall										
Model 1	0.123	0.102	0.021	0.701	0.127	0.024	0.095	0.087	0.018	0.733
Model 2	0.142	0.058	0.024	0.658	0.113	0.049	0.068	0.224	0.022	0.685
*APOE4*-negative										
Model 1	0.145	0.066	0.019	0.735	0.170	0.005	0.105	0.067	0.026	0.638
Model 2	0.170	0.036	0.019	0.746	0.177	0.007	0.084	0.163	0.035	0.549
*APOE4*-positive										
Model 1	0.008	0.972	0.150	0.347	−0.101	0.511	0.104	0.562	−0.118	0.467
Model 2	−0.104	0.516	−0.032	0.781	−0.111	0.348	−0.102	0.443	−0.030	0.785

Abbreviations: MAC, mid-arm circumference; CC, calf circumference; WC, waist circumference; *APOE4*, apolipoprotein E ε4 allele; EMS, episodic memory score; TS, total score of the Consortium to Establish a Registry for Alzheimer’s Disease; VRS, vascular risk score. The first model included age, sex, *APOE4*, education, clinical diagnosis, VRS, and BMI as covariates; the second model included those covariates plus GDS score, PASE score, annual income, alcohol intake, smoking, albumin, fasting glucose, and HDL- or LDL-cholesterol.

## Data Availability

The study data are not freely accessible because the IRB of the Hallym University Dongtan Sacred Heart Hospital prevents public sharing of such data for privacy reasons. However, the data are available on reasonable request after IRB approval. Requests for data access can be submitted to an independent administrative coordinator by e-mail (yoon4645@gmail.com).

## Supplementary Materials

The following supporting information can be downloaded at: https://www.mdpi.com/article/10.3390/ijms26125831/s1.
