# Supplementary material for: Body Circumference and Cognitive Function: Role of Apolipoprotein E ε4 in the Elderly
Source: Int J Mol Sci. 2025 Jun 18;26(12):5831. doi: 10.3390/ijms26125831 (PMC12193496; doi:10.3390/ijms26125831)
Supplement: Supplementary file 1 [file ijms-26-05831-s001.zip › ijms-3627488-supplementary.pdf]

Supplemental materials

Contents

- **Table S1.** Results of multiple linear regression analyses that included interaction terms for the association between body circumference and sex in predicting cognition
- **Table S2.** Results of the multiple linear regression analyses of the association between body circumference and cognition according to sex subgroup

Table S1. Results of multiple linear regression analyses that included interaction terms for the association between body circumference and sex in predicting cognition.

	MAC		CC		MAC/WC		CC/WC		MAC/CC	
	β	*P*	*β*	*P*	*β*	*P*	*β*	*P*	*β*	*P*
**EMS**										
BC	0.024	0.796	-0.043	0.619	0.341	<0.001	0.277	<0.001	0.043	0.599
Sex	-0.933	0.108	-0.790	0.158	-0.077	0.893	0.130	0.829	-0.050	0.911
BC ×Sex	0.986	0.098	0.856	0.133	0.091	0.875	-0.105	0.862	0.099	0.824
**NMS**										
BC	0.029	0.753	0.111	0.023	0.228	0.005	0.325	<0.001	-0.078	0.340
Sex	-1.025	0.075	-0.367	0.249	0.015	0.980	-0.442	0.444	0.239	0.586
BC ×Sex	1.155	0.051	0.475	0.143	0.091	0.878	0.549	0.345	-0.113	0.798
**TS**										
BC	0.062	0.502	-0.014	0.825	0.327	<0.001	0.281	<0.001	0.024	0.766
Sex	-0.819	0.156	-0.600	0.133	0.032	0.956	-0.170	0.776	0.128	0.773
BC ×Sex	0.884	0.136	0.572	0.159	0.006	0.991	0.220	0.714	-0.058	0.896

Abbreviations: BC, body circumference; MAC, mid-arm circumference; CC, calf circumference; WC, waist circumference; APOE4, apolipoprotein ε4 allele; EMS, episodic memory score; NMS, non-memory score; TS, total score of the Consortium to Establish a Registry for Alzheimer's Disease.

To explore the moderating effects of sex on the associations between BC and cognition, i.e., EMS, NMS, and TS, the multiple linear regression analyses were performed including two-way interaction terms between BC and cognition as additional independent variables.

Table S2. Results of the multiple linear regression analyses of the association between body circumference and cognition according to sex subgroup.

	MAC		CC		MAC/WC		CC/WC		MAC/CC	
	β	*P*	*β*	*P*	*β*	*P*	*β*	*P*	*β*	*P*
**EMS**										
Female										
Model 1	0.343	**0.004**	-0.034	0.484	0.344	**<0.001**	0.038	0.487	0.049	0.348
Model 2	0.320	**0.002**	-0.072	0.154	0.109	**0.039**	-0.013	0.812	0.082	0.102
Male										
Model 1	0.379	**0.002**	0.028	0.786	0.351	**0.007**	0.056	0.592	0.111	0.332
Model 2	0.403	**0.006**	0.029	0.793	0.254	**0.026**	0.011	0.920	0.123	0.329
**NMS**										
Female										
Model 1	-0.058	0.558	0.415	**<0.001**	0.011	0.884	0.348	**<0.001**	-0.106	0.132
Model 2	-0.073	0.441	0.553	**<0.001**	0.032	0.654	0.172	**0.020**	-0.065	0.337
Male										
Model 1	0.319	0.093	0.403	**0.001**	0.152	0.304	0.402	**0.001**	-0.103	0.443
Model 2	0.332	0.084	0.539	**<0.001**	0.099	0.563	0.434	**<0.001**	-0.203	0.162
**TS**										
Female										
Model 1	0.116	0.052	-0.015	0.800	0.328	**<0.001**	0.052	0.391	0.009	0.874
Model 2	0134	**0.024**	-0.061	0.288	0.134	**0.019**	-0.003	0.957	0.044	0.438
Male										
Model 1	0.345	**0.039**	0.179	0.122	0.323	**0.013**	0.219	0.059	-0.002	0.987
Model 2	0.458	**0.018**	0.105	0.405	0.263	**0.043**	0.211	0.084	-0.046	0.752

Abbreviations: MAC, mid-arm circumference; CC, calf circumference; WC, waist circumference; APOE4, apolipoprotein E ε4 allele; EMS, episodic memory score; TS, total score of the Consortium to Establish a Registry for Alzheimer's Disease; VRS vascular risk score.

The first model included age, APOE4, education, clinical diagnosis, VRS, and BMI as covariates; the second model included those covariates plus GDS score, PASE score, annual income, alcohol intake, smoking, albumin, fasting glucose, and HDL- or LDL-cho lesterol.
